# Productivity costs of schizophrenia spectrum and other psychotic disorders by friction cost and human capital methods: The Northern Finland Birth Cohort 1966

**DOI:** 10.1007/s00127-024-02652-y

**Published:** 2024-03-22

**Authors:** Tuomas Majuri, Iiro Nerg, Sanna Huikari, Ina Rissanen, Erika Jääskeläinen, Jouko Miettunen, Marko Korhonen

**Affiliations:** 1 Research Unit of Population Health, Faculty of Medicine, University of Oulu, P.O.BOX 5000, Oulu, Finland; 2 Medical Research Center Oulu, Oulu University Hospital and University of Oulu, Oulu, Finland; 3 Department of Psychiatry, Oulu University Hospital, Oulu, Finland; 4 Department of Economics, Accounting and Finance, University of Oulu, Oulu, Finland; 5 Julius Center for Health Sciences and Primary Care, University Medical Center Utrecht and Utrecht University, Utrecht, the Netherlands

**Keywords:** Schizophrenia, Psychosis, Indirect costs, Productivity cost, Follow-up, Cohort study

## Abstract

**Purpose:**

Psychotic disorders are associated with substantial productivity costs; however no previous studies have compared these between schizophrenia spectrum (SSD) and other psychotic disorders (OP). The human capital method (HCM) and the friction cost method (FCM) are the two most common approaches to assess productivity costs. The HCM focuses on employees’ perspectives on the costs, whereas the FCM demonstrates employers’ perspectives. Studies comparing these methods when estimating the productivity costs of psychoses are lacking.

**Methods:**

Utilizing the Northern Finland Birth Cohort 1966 with linkages to national registers, we compared the adjusted productivity costs of SSD (*n* = 216) and OP (*n* = 217). The productivity costs were estimated from ages 18 to 53 including projections to statutory retirement age using the FCM and HCM.

**Results:**

When estimated via the HCM, productivity losses were higher for SSD (€193,940) than for OP (€163,080). However, when assessed using the FCM, costs were significantly lower for SSD (€2,720) than for OP (€4,430). Productivity costs varied by sex and various clinical and occupational factors.

**Conclusion:**

This study highlights how productivity costs vary by psychosis diagnosis. These differences should be noted when planning interventions. The low FCM estimates indicate the need of interventions before or during the early phases of psychoses. From a societal perspective, interventions are needed, particularly for those with highest HCM productivity losses, such as males with SSD. Besides psychiatric services, the roles of social services, employment agencies and occupational health care should be considered when helping individuals with psychoses to working life.

## Introduction

Many studies have assessed the societal costs of schizophrenia, highlighting the substantial economic burden of the disorder [1]. However, no previous studies have compared the productivity costs of schizophrenia with those of other psychoses (OP). Furthermore, studies estimating productivity costs due to psychoses from employees’ and employers’ perspectives are almost nonexistent.

The costs of illness (COI) can be divided among direct health care costs, direct non-health care costs and indirect costs [2]. Indirect costs are the costs of losses in productivity resulting from the morbidity and premature mortality of affected persons [3]. Productivity costs can be defined as the economic value of forgone production that is associated with the loss of paid and unpaid work [4]. Productivity losses are the single most financially burdensome component of the societal costs of schizophrenia, accounting for 32–83% of the total costs across studies [1].

The human capital method (HCM) and the friction cost method (FCM) are the two most widespread approaches employed to assess productivity costs [1, 5]. The HCM focuses on employees’ perspectives on the costs, whereas the FCM demonstrates employers’ perspectives. The HCM aims to estimate production loss potential over the employees’ expected remaining working life by multiplying the loss of earnings for different sex and age groups by the corresponding number of individuals in that group [4, 5]. The HCM assumes that had the disease been avoided, the individual would have remained alive, employed, and healthy until retirement [6]. The FCM assumes that individuals who stop working due to illness will be replaced by someone who was previously unemployed and measures the productivity loss during that period [4, 5, 7]. Certain adjustments to compensate for the impact of hidden assumptions in the models have been suggested [6]. These adjustments include considering the proportion of disability-free life expectancy, unemployment coefficients and overall coefficient of work ability decline in the HCM as well as the annual unemployment rate and the proportion of employed jobseekers in the FCM.

Whether the FCM or HCM is used when estimating productivity costs of psychoses varies between studies [1, 3, 8, 9]. Each method assesses costs from a different perspective, making comparisons between the methods difficult. When estimating the productivity costs of schizophrenia, only in one previous sample have both the FCM and HCM been used [10]. However, the study only included productivity costs due to premature mortality and assessed productivity costs for only one year [10]. The study found the productivity costs assessed by using the HCM to be significantly higher than those estimated by the FCM [10]. Likewise, a recent review reported productivity losses in schizophrenia to be 70-times higher in studies using the HCM when compared to studies using the FCM [9].

Most COI studies on psychoses do not clearly report diagnostic categories using the relevant diagnostic classification codes [1], nor do they distinguish between schizophrenia spectrum disorders (SSD) and OP, despite the different nature of these disorders. Similarly, despite existing differences in psychosocial functioning [11], labour market attachment [12], and rates of disability pensions [13] between SSD and OP, no previous studies have focused on differences in productivity costs between these disorders.

This study aimed to compare the productivity costs of SSD and OP in a Finnish psychosis sample. Altogether, using prospective data drawn from a large population-based birth cohort with individual-level linkages to various national registers, we specifically aimed to compare estimates of productivity costs using the FCM and HCM among individuals with SSD and OP.

## Methods

### Sample

The study was based on the Northern Finland Birth Cohort 1966 (NFBC1966) [14], which is an unselected, prospective, general population sample comprising information on 12,058 live-born children in the provinces of Oulu and Lapland, with expected dates of birth in 1966. The cohort members have been followed up with data collection at different ages, including linkages with national register data [15].

### Case detection

All cohort members with SSD or OP diagnoses before 2020 were searched from numerous national registers: the Care Register for Health Care (CRHC) [16], the Register of Primary Health Care Visits (2011–) [16], the Finnish Centre for Pensions (FCP) (1974-) [17], and The Social Insurance Institution of Finland (SII) and included in the study [18]. The diagnostic categories based on different versions of the International Classification of Diseases (ICD) and used in the study were SSD (ICD-8: 2950–2959, 297; ICD-9: 2950–2959, 297; ICD-10: F20, F22, F24, F25) and OP (ICD-8: 2960–2969, 2980–2983, 2988, 2989, 299; ICD-9: 2961E, 2962E, 2963E, 2964E, 2967, 2988, 2989; ICD-10: F23, F28, F29, F302, F312, F315, F323, F333). This definition was based on the classification used in the previous studies of the NFBC1966 [12, 19, 20] and considering the chronic nature of schizophrenia, schizoaffective disorder and delusional disorder compared to other, non-schizophrenic psychoses.

When determining the psychosis diagnosis, we used a hierarchical system to deal with subjects with multiple diagnoses or movement between diagnostic categories over the follow-up period. In this system, the life-time psychosis diagnosis was the disorder that had the highest position in the hierarchy based on severity (SSD, OP). This hierarchy has been used in previous studies of the NFBC1966 [12].

We identified 216 subjects with SSD and 217 with OP; together, these subjects formed the final sample (*n* = 433).

### Estimation of productivity costs

We used model specifications of both FCM and HCM cost estimates in a Finnish psychosis sample [6]. These models are similar to those used in previous research on the NFBC1966 [21]. To evaluate productivity costs, we linked various population-level data with individual-level data on incident psychoses, income, work absence, and mortality. Productivity costs were assessed separately for SSD and OP, starting from the date of the first occurrence of a diagnosis of any psychotic disorder in the register data.

### Measures

#### Population-level data

Multiple sources were used to provide annual population-level data. A detailed description of the data collection method has previously been described in detail [21].

The data on life expectancy (available from 1986 to 2021, including projections from 2021 onward) and unemployment rate (from 1997 to 2020) were obtained from Statistics Finland [22]. Information on average disability-free life expectancy (available from 1996 to 2021) was obtained from the statistics of the Finnish Institute for Health and Welfare [16] and estimated as the proportion of the total population of the same age receiving disability pensions. The data on the proportion of employed jobseekers (2006–2021) were calculated based on information from Statistics Finland [22]. The data on vacancy periods (2006–2021) according to occupational class were gathered from the statistics of the Ministry of Economic Affairs and Employment [23].

The median wages of specific occupational groups in the Finnish population were obtained from the FOLK personal data modules [24] and used as a proxy for the value of daily productivity. Information on gross domestic product (GDP) growth from the World Bank [25] and the monetary value multiplier from Statistics Finland [22] were used to discount all monetary values (of daily productivity) into 2021 values.

#### Individual-level data

Various national registers were used to provide individual-level data for the sample.

Illness onset, meaning the age when the diagnosis of any psychotic disorder occurred for the first time in the register data, was defined using the CRHC, the SII registers of reimbursable medicines, the FCP register, and Finnish outpatient registers.

Registers of the SII (until 2016) [18] and the FCP (until 2019) [17] were used to collect data on sick leave and disability pensions. Annual data on educational level (until 2019), occupational and socioeconomic status (until 2018) and information on dates and causes of death (until February 2023) were gathered from the registers of Statistics Finland [22].

The duration of work absence before and after the onset of psychosis was evaluated based on the starting and ending dates of sick-leave periods, disability pensions and death dates. The data on sick leave, disability pensions, and deaths from the registers of the SII (until 2016) and the FCP (until 2019) were used to calculate the total duration of work absence since the individual’s 18th birthday. If the person had been granted a permanent disability pension or had died before 2019, the person was assumed to be absent from work until retirement age (65 years).

Information on perceived work ability (scaled from 0 to 10) was based on the NFBC1966 31-year and 46-year follow-up questionnaires. An index of overall work ability decline (WAD) was calculated for the study population.

### Missing data

Information on socioeconomic status was missing for 3% of individuals with psychosis when analysing the characteristics of the sample and when presenting the productivity cost estimates by socioeconomic status. These individuals were included in the FCM and HCM analyses. The information on socioeconomic status was not used when calculating the productivity costs.

### Statistical analyses

#### Background characteristics and differences in productivity cost estimates

The background variables in the SSD and OP groups were presented using cross-tabulation (categorical variables) and medians with interquartile ranges (continuous variables). Differences in background characteristics were tested with the chi-square test (categorical variables) and ANOVA (continuous variables). Differences in the FCM and HCM cost estimates were tested with a *t*-test. *P* values < 0.05 were considered statistically significant. All tests were two-tailed. The statistical analyses were conducted using R version 4.0.3.

#### Friction cost method

Using the FCM, we assessed productivity costs as the duration of absence from work after a psychosis onset multiplied by the value of daily production if the period of absence was shorter than the estimated friction period. In cases where the duration of absence was longer than the friction period, we used the friction period in calculations instead of the actual absence duration. Considering earlier literature [6], we conducted an adjusted model for productivity costs. The occupation-specific median wages of Finnish people born in 1965 − 1967 were used to monetarise the value of production. The friction period was annually estimated to be the occupation-specific average vacancy period increased by 60 days, which was assumed to be the time employers needed to place a vacancy and to train a replacement for an absent worker [26]. The model was further adjusted for the length of the vacancy chain (LVC) [6]. The estimate for the LVC was as follows:

$${\rm{LVC}}\,{\rm{ = }}\,\alpha \,{\rm{ \times }}\,\left( {{\rm{1}}\, - u} \right)\,{\rm{/}}u$$

where *α* is the proportion of employed jobseekers and *u* is the annual unemployment rate.

#### Human capital method

Using the HCM, we calculated productivity costs as days absent from work after psychosis onset due to sick leave, disability pension, or death until the statutory retirement age, multiplied by the value of daily production each year. Considering earlier literature [6], we conducted an adjusted model for productivity costs. The median wages of Finnish people born in 1965 − 1967 were used as a base to monetarise the value of production. First, we adjusted these values by sex and occupation. Then, to acknowledge future labour force participation, we further adjusted the model by considering the proportion of disability-free life expectancy (PLDF), unemployment coefficients, and overall coefficients of work ability decline. The productivity costs in the HCM were calculated as follows:

$${\rm{PC}}\,{\rm{ = }}\,{\rm{wage}}\,{\rm{ \times }}\,t\,{\rm{ \times }}\,{\rm{PLDF}}\,{\rm{ \times }}\,\left( {{\rm{1}} - u} \right)\,{\rm{ \times }}\,{\rm{WAD,}}$$

where *t* is the time of absence in days (until the retirement age), PLDF is the disability-free life expectancy/life expectancy, *u* is the national annual unemployment rate and WAD is the estimated coefficient for the overall decline in work ability.

## Results

### Characteristics of the sample

More individuals with OP (15%) than with SSD (10%) were working at psychosis onset (*p* = 0.038) (Table 1). At the age of 46, there were significantly more pensioners with SSD (67%) than with OP (37%). The mean age of psychosis onset was 31 years for individuals with SSD, and 38 years for those with OP (*p* < 0.001).

**Table 1:** Characteristics of the sample

Variable	Schizophrenia spectrum disorder (*n* = 216)	Other psychosis (*n* = 217)	Total (*n* = 433)	*p*-value (SSD vs. OP)
**Sex, n (%)**				0.225
Male	126 (58.3)	114 (52.5)	240 (55.4)	
Female	90 (41.7)	103 (47.5)	193 (44.6)	
**Educational level, n (%)** ^**a**^				0.373
Basic	50 (23.1)	47 (21.7)	97 (22.4)	
Secondary	117 (54.2)	108 (49.8)	225 (52.0)	
Tertiary	49 (22.7)	62 (28.6)	111 (25.6)	
**Work status at psychosis onset, n (%)**				0.038
Working	21 (9.7)	33 (15.2)	54 (12.5)	
Working and benefit period	27 (12.5)	42 (19.4)	69 (15.9)	
Benefit period	87 (40.3)	78 (35.9)	165 (38.1)	
Not working or benefit period	81 (37.5)	64 (29.5)	145 (33.5)	
**Working one year after diagnosis, n (%)**				0.075
No	106 (49.1)	88 (40.6)	194 (44.8)	
Yes	110 (50.9)	129 (59.4)	239 (55.2)	
**Socioeconomic status, n (%)** ^**b**^				< 0.001
Skilled worker	15 (7.2)	45 (21.3)	60 (14.3)	
Pensioner	139 (66.5)	77 (36.5)	216 (51.4)	
Other	55 (26.3)	89 (42.2)	144 (34.3)	
**Age at onset of psychosis, Mean (SD)**	30.8 (9.3)	38.0 (9.8)	34.3 (10.2)	< 0.001
**Age at onset of psychosis, n (%)**				< 0.001
Under 25	63 (29.2)	27 (12.4)	90 (20.8)	
Over 25	153 (70.8)	190 (87.6)	343 (79.2)	

^a^At 2019, ^b^At 2012

*SSD* schizophrenia spectrum disorder, *OP* other psychosis

### Productivity costs

The average productivity costs determined using the FCM were significantly lower in SSD (€2,720) than in OP (€4,430) (Table 2). Using the HCM, productivity costs were €193,940 in SSD and €163,080 in OP. The expected productivity costs until the retirement age (in 2031) were €302,250 in SSD and €267,530 in OP, according to the HCM.

**Table 2:** Observed and expected productivity cost estimates by sex and psychosis diagnosis with FCM and HCM

	**FCM**					
	**Years 1984–2019 (Observed)**			**Years 1984–2031 (Expected)**		
	All*	Male*	Female*	All*	Male*	Female*
Schizophrenia spectrum disorder	€2,720	€2,860	€2,520	€2,720	€2,860	€2,520
Other psychosis	€4,430	€4,290	€4,590	€4,480	€4,290	€4,680
All diagnoses	€3,580	€3,540	€3,630	€3,600	€3,540	€3,670
	**HCM**					
	**Years 1984–2019 (Observed)**			**Years 1984–2031 (Expected)**		
	**All**	**Male**	**Female**	**All**	**Male**	**Female**
Schizophrenia spectrum disorder	€193,940	€208,030	€174,220	€302,250	€317,070	€281,500
Other psychosis	€163,080	€175,070	€149,810	€267,530	€284,880	€248,330
All diagnoses	€178,470	€192,370	€161,190	€284,850	€301,780	€263,800

*p-value < 0.05 between schizophrenia spectrum disorder and other psychosis

*FCM* friction cost method, *HCM* human capital method

The productivity costs determined using the FCM were significantly higher in both males and females with OP (€4,290 − 4,590) than among those with SSD (2,520 − 2,860) (Table 2). When estimated using the HCM, the average productivity costs were higher in both males (€208,030) and females (€174,220) with SSD than in males (€175,070) and females with OP (€149,810).

Overall, in psychoses, average productivity costs determined using the FCM were within the same range for males (€3,540) and females (€3,630) (Table 3). However, using the HCM, productivity costs were higher among males (€192,370) than among females (€164,720). A higher onset age of psychosis was associated with higher FCM costs but lower HCM costs. A higher educational level produced higher productivity costs in both SSD and OP using both methods. Productivity costs were higher if the person was working or in a benefit period at psychosis onset when estimated using both the FCM and the HCM. The FCM cost estimates were the highest among skilled workers, whereas pensioners had the highest costs when determined using the HCM. Returning to work one year after the psychosis diagnosis was associated with higher productivity costs according to both methods.

**Table 3:** Adjusted FCM and HCM estimates in 1984–2019

Variable	FCM costs			HCM costs		
	All	Schizophrenia spectrum disorder	Other psychosis	All	Schizophrenia spectrum disorder	Other psychosis
**Sex**						
Male	€3,540	€2,860	€4,290	€192,370	€208,030	€175,070
Female	€3,630	€2,520	€4,590	€161,190	€174,220	€149,810
**Age at onset of psychosis**						
Under 25	€810	€610	€1,300	€189,140	€191,880	€182,750
Over 25	€4,300	€3,590	€4,880	€175,670	€194,790	€160,280
**Educational level** ^**a**^						
Basic or below	€2,390	€1,550	€3,270	€154,850	€174,890	€133,520
Secondary	€3,100	€2,360	€3,890	€179,590	€197,030	€160,690
Tertiary	€5,590	€4,750	€6,260	€196,860	€205,990	€189,640
**Work status at psychosis onset**						
Working	€5,250	€5,370	€5,180	€183,620	€188,480	€180,530
Working and benefit period	€5,050	€3,750	€5,880	€183,880	€248,780	€142,160
Benefit period	€3,310	€2,380	€4,340	€194,960	€207,820	€180,610
Not working or benefit period	€2,560	€2,040	€3,220	€155,220	€162,170	€146,430
**Socioeconomic status** ^**b**^						
Skilled worker	€6,300	€6,340	€5,110	€100,400	€123,820	€92,590
Pensioner	€2,540	€1,380	€3,830	€214,360	€202,020	€236,630
Other	€4,230	€2,930	€4,600	€161,550	€198,520	€138,710
**Working one year after diagnosis**						
No	€3,180	€2,410	€4,100	€146,710	€146,890	€146,490
Yes	€3,900	€3,010	€4,660	€204,260	€239,280	€174,390

^a^At 2019, ^b^At 2012

*FCM* friction cost method, *HCM* human capital method

## Discussion

### Main findings

To our knowledge, this is the first study to compare productivity costs between SSD and OP estimated using both the FCM and the HCM. When assessed using the FCM, productivity costs were significantly higher in OP; however when using the HCM, costs were higher in SSD. The FCM produced lower cost estimates compared to the HCM. Productivity costs varied by sex and different sociodemographic and clinical factors.

### Comparison to previous studies

As suggested [1], we considered different psychosis diagnoses and compared productivity costs between SSD and OP. We found higher productivity costs in SSD than in OP when estimated using the HCM but lower costs in SSD than in OP when assessed using the FCM. SSD has been associated with lower psychosocial functioning [11], poorer labour market attachment [12], lower levels of education [11, 13] and higher rates of disability pensions when compared to OP [11]. Individuals with higher levels of socioeconomic class and education are more likely to return to work, producing lower costs using the HCM, but since the return to work does not happen inside the friction period, the wage differences override the differences in the duration of work absence targeted by the FCM [21]. A higher onset age for OP than SSD also has an effect in the HCM, as in long-term studies, individuals with OP may spend more years working before their work ability is reduced, leading to the accumulation of productivity losses after the onset of psychosis. A recent meta-analysis found that the median age at the illness onset of schizophrenia-spectrum and primary psychotic disorders is 25 years and that age at the onset of OP is typically higher than in SSD [27]. The mean age at the psychosis onset in our study was relatively high (31 for SSD and 38 for OP). This characteristic could be partly explained by using register information in defining the age of onset in this study since the registers indicate the start of treatment instead of the onset of psychotic symptoms. Furthermore, the use of a population-based sample born in 1966 and with a very long follow-up may have led to higher onset ages compared to studies with younger patients and shorter follow-ups.

Our results align with a previous study that reported substantially higher productivity costs in schizophrenia obtained by the HCM than those estimated by the FCM [10]. Studies involving disorders with a permanent work disability and substantial mortality among working-age adults have been found to yield higher productivity cost estimates if the HCM is used [4], as also seen in psychotic disorders.

The HCM has been criticised for overestimation of productivity losses due to measuring lost potential values instead of actual productivity [4]. Compared to previous studies using the HCM [1], our estimates of productivity costs in SSD until retirement age produced higher figures. Our estimates in SSD using the FCM are not well comparable to previous studies, since these studies have also included caregiver costs or calculated only mortality costs using the FCM and applied the HCM when assessing morbidity costs, thus leading to higher cost estimates [1].

Compared to other disorders with a high degree of disability such as stroke [21], the adjusted lifetime productivity costs by HCM in psychoses are even higher, highlighting the status of psychoses as one of the disorders with the highest societal burden already seen in early adulthood. However, when assessed via the FCM, the lifetime productivity costs of psychoses are somewhat lower compared to those of stroke, reflecting the poor occupational outcome of psychoses not only after but also before the onset of psychosis.

In the HCM, the productivity costs were higher among males than females. The FCM produced similar costs for males and females. These findings are in line with the sex-specific average number of benefit days in this study. Gender gaps in the labour market persist throughout the world [28]. In general, females typically have more sickness absences compared to males [29]. In psychoses, there is no consensus regarding sex differences in employment levels [30]. Some studies have revealed better occupational outcomes for females than for males with schizophrenia [31]. However, other studies have suggested better outcomes for males in terms of paid employment in some regions of the world [32]. A previous study in the NFBC1966 found that compared to males, females with SSD were more likely to be pensioners, whereas in OP, the rate of pensioners was identical across sexes [12]. Females with OP have been found to have higher educational levels and socioeconomic status compared to females with SSD in the NFBC1966 [12]; thus, the probability of returning to work in the long run but not during the friction period may be more common for females with OP, leading to sex differences between the HCM and FCM. There also may be difference in the duration of untreated psychosis between males and females. However, sex has not been associated with the duration of untreated psychosis in the NFBC1966 [33]. Furthermore, shorter life expectancy in males than in females with psychotic disorders [34] has an effect, as males may not reach the statutory retirement age as often as females. All productivity cost estimates based on wages may value the health of males over that of females, of high-income earners over that of low-income earners and of workers over non-workers [21].

Productivity costs in the FCM and HCM were higher if a person was working or in a benefit period at psychosis onset and working after diagnosis. Individuals with psychosis often encounter unemployment and part-time work already during early adulthood, and unemployment tends to continue later throughout their lives [12]. For this reason, productivity does not have time to rise before the onset of psychosis, and median wages will remain low in most cases. A few individuals may spend years at work before the illness onset and immediately after the diagnosis, giving them more time to increase productivity before long-term disability. Productivity costs assessed using the FCM were the highest among skilled workers. This may be explained by skilled workers’ longer occupation-specific friction periods and higher median wages.

Individuals with SSD often experience disturbances in cognition and different fields of functioning already before and later during the prodromal phase of psychosis [35]. These individuals typically attain low general academic achievement scores, are unlikely to enter higher education [36] and present with weak labour market attachment during working life [12]. The reduced pre-morbid cognitive functioning is also seen in OP but on a smaller scale [37]. Compared to those with SSD, individuals with OP have a somewhat better level of psychosocial functioning in terms of being employed or studying, having children and independent living [11]. First-episode psychosis typically occurs in late adolescence or early adulthood and leads to hospitalisation [38]. The average time between the first hospitalisation and disability pension in SSD varies between 1 and 4 years [12]. For some individuals with SSD or OP, it is possible to return to the labour market after being on a disability pension [19].

In terms of reporting productivity losses in psychoses, further studies should be sufficiently specific to produce accurate information for comparison between different studies and policymaking. For example, recommendations for best practices for conducting COI studies on schizophrenia have been suggested [1].

### Strengths and limitations

Our general population birth cohort sample offers a comprehensive picture covering all branches of occupations and the economy when estimating the productivity costs of SSD and OP. Whereas most of the previous studies have assessed productivity costs during a period of one to a few years, we were able to analyse costs longitudinally from the ages of 18 to 53 years, including projections until statutory retirement age. The use of high-quality register data enabled us to study the productivity costs of psychoses very specifically among individuals with different sociodemographic backgrounds and to note important adjustments. By using data from multiple national registers in the case detection phase, our results fully describe the disability-related productivity costs in all persons with SSD and OP in this population. One of the study’s strengths was the opportunity to study SSD and OP separately instead of studying psychoses in general. Previous studies from countries with labour market circumstances similar to Finland [39] have used only the HCM when estimating the costs of SSD [40–42]. We were able to assess productivity costs due to psychoses for the first time in Finland, a country with one of the highest prevalences of psychotic disorders worldwide [13, 43], using both the FCM and HCM. Our findings are derived from a Nordic welfare country which provides access to education, health care and social security for all citizens, so the results are most generalisable to countries with similar labour market circumstances.

The study has certain limitations. Due to the long follow-up, period effects should be noted. The use of three diagnostic classifications over decades and differences in diagnostic practices may have resulted in variations in the prevalence of different diagnoses and differences in the grounds for granting disability pensions. The diagnostic categories used in the study were based on different versions of the ICD. Another diagnostic classification that is widely used in some other studies is the Diagnostic and Statistical Manual of Mental Disorders (DSM). The main difference in psychosis diagnosis between these diagnostic classifications is that in ICD-10, the duration of psychotic symptoms must have persisted at least one month whereas in DSM-5, symptoms must have been for at least six months. Since this, the prevalence of psychoses may be higher in studies using ICD. Moreover, studies using the ICD may include individuals with milder symptoms and better functioning which may lead to more favourable outcomes, also in our study. The reliability of the schizophrenia diagnoses in Finnish registers is good [44], also among individuals in the NFBC1966 [45]. However, there may be slight differences in diagnostic practices between clinicians and at different times. There was a shortage of psychiatrists in the 1990s in northern areas of Finland and some diagnoses were made by non-specialist physicians [45]. Furthermore, the number of psychiatric inpatient services in Finland has been decreasing for several decades and there has been a tendency for shorter periods at hospitals [46]. For these reasons, clinicians at different times may have underestimated the prevalence of schizophrenia diagnoses among all psychotic diagnoses [45] and some individuals with OP might fulfil the criteria of SSD also in this study.

We used broad definitions for SSD and OP in line with the previous studies in the NFBC1966. However, there are no standard definitions for SSD and OP and definitions for these vary between studies, diagnostic systems and at different times [47]. For example, the criteria for the duration of symptoms of schizophrenia have changed between different versions of ICD. Due to very long-term follow-up in our study, some individuals diagnosed with schizophreniform disorders before ICD-10 would nowadays fulfil the criteria for schizophrenia. There is overlap in symptoms, neurocognition, and risk factors among psychotic disorders classified within SSD and OP leading to challenges when using categorical approaches for research purposes [48]. There is considerable variation in the classification of psychoses across studies. For example, in some studies, all psychotic disorders (F20-F29) are classified as schizophrenia [49]. However, previous studies have found significant variation in occupational outcomes between SSD and OP [11–13], and our purpose was to focus on differences in the economic burden arising from this variation. These issues may lead to uncertainty in our results comparing SSD and OP, and the results of our study are best comparable to studies using similar classification than ours.

We assessed productivity costs starting from the first occurrence of a diagnosis of any psychotic disorder in the register data. However, the registers indicate the start of treatment instead of the start of actual symptoms. We note that we were not able to consider the prodromal phases of psychoses, which may strongly determine the productivity estimates [50]. The small sample sizes, with respect to analysing SSD and OP separately, limit the statistical power of our study. Likewise, the small sample sizes limit the detailed examination of the subgroups of psychotic diagnoses, such as schizophrenia (F20), delusional disorders (F22) and affective psychoses. In the future, it would be important to assess the productivity costs of different psychosis diagnoses in larger samples to replicate the conclusions of this study.

### Clinical implications

Compared to SSD, productivity losses in OP were somewhat higher according to the FCM and lower when using the HCM, which highlights the differences in labour market attachments between psychotic disorders.

The significant productivity costs calculated, irrespective of the method used, emphasise the poor occupational outcomes in SSD and OP. Different kinds of interventions have been suggested to prevent long-term exclusions from the labour market. From a societal perspective, interventions are particularly needed for those with the highest HCM productivity losses, such as males with SSD. As these persons are not typically in the labour market, the role of sectors other than psychiatric services should be considered. For example, social services and employment agencies should be acknowledged already at an early stage, and increased co-operation between social and psychiatric services should be developed.

Differences in occupational outcomes between psychotic disorders should be noted when planning rehabilitation services and more studies on the vocational rehabilitation of psychoses other than SSD are needed. Compared to SSD, individuals with OP are typically more attached to working life before psychosis onset. Thus, it would be important to invest in and add knowledge on psychoses in occupational health care services and workplaces that play a remarkable role in helping individuals back to working life.

As the return to work in psychoses is unlikely to happen within the friction period due to a lack of services and complex sick-leave systems, productivity losses could be reduced by investing in early intervention services that help individuals regain employment after first-episode psychoses [51]. However, development of new interventions is needed, as the effectiveness of early intervention services may not remain in long-term follow-ups [52]. The Individual Placement and Support (IPS) approach has been found effective in improving employment outcomes among individuals with psychoses [53]. The IPS should be considered not only for individuals with first-episode psychoses but also for individuals with multiple episodes and longer histories of psychotic disorders. However, the IPS has been found less effective in European studies compared to non-European studies and for people with a higher symptom severity compared to a low symptom severity [53]. Moreover, long-term findings on the effectiveness of the IPS are still lacking. The relatively low FCM cost estimates in psychoses indicate that interventions should be started not only after the onset but also already during the prodromal phase of psychosis, as individuals with psychoses are unlikely to enter working life or reach high socioeconomic status before the diagnosis [54].

## Conclusion

SSD and OP entail remarkable productivity costs. This study highlights the differences in productivity costs according to psychosis diagnosis and different sociodemographic factors while offering important information on the indirect costs of psychoses for clinicians and decision makers. The relatively low FCM estimates indicate the need for interventions at the early stages of psychotic disorders, whereas the high HCM estimates highlight the need for interventions for individuals with the highest productivity losses, such as males with SSD. Besides psychiatric services, the role of social services, employment agencies and occupational health care should be noted when helping individuals with psychotic disorders to working life.

## Data Availability

NFBC data is available from the University of Oulu, Infrastructure for Population Studies. Permission to use the data can be applied for research purposes via electronic material request portal. In the use of data, we follow the EU general data protection regulation (679/2016) and Finnish Data Protection Act. The use of personal data is based on cohort participants written informed consent at their latest follow-up study, which may cause limitations to its use. Please, contact the NFBC project center (NFBCprojectcenter@oulu.f) and visit the cohort website (www.oulu.f/nfbc) for further information.
